# Supplementary material for: CoNECo: a Corpus for Named Entity recognition and normalization of protein Complexes
Source: Bioinform Adv. 2024 Aug 20;4(1):vbae116. doi: 10.1093/bioadv/vbae116 (PMC11474106; doi:10.1093/bioadv/vbae116)
Supplement: vbae116_Supplementary_Data [file vbae116_supplementary_data.pdf]

Supplementary Material
"CoNECo: A Corpus for Named Entity recognition and normalization of protein Complexes"

Katerina Nastou[1], Mikaela Koutrouli[1], Sampo Pyysalo[2], Lars Juhl Jensen[1]

[1]Novo Nordisk Foundation Center for Protein Research, University of Copenhagen, Blegdamsvej 3, 2200, Copenhagen, Denmark and
[2]TurkuNLP Group, Department of Computing, University of Turku, Turku, Finland

Supplementary Table 1: Normalization errors for Jensenlab tagger run on the CoNECo test set.

PubMed ID	annotated entity	tagged entity	annotated normalization	tagger normalization	comment
23499533_3	cohesin	cohesin loading	GO:0008278	GO:0032116	cohesin loading complex is a narrow synonym for SMC complex, and is tagged as the left-longest entity
16209941	SCF(Skp2) ubiquitin ligase	SCF	GO:0097669	GO:0019005	the more general SCF has been tagged and normalized
16209941	SCF(Skp2)	SCF	GO:0097669	GO:0019005	the more general SCF has been tagged and normalized
16209941	SCF(Skp2)	SCF	GO:0097669	GO:0019005	the more general SCF has been tagged and normalized
20427570_13	Complex III	Complex III	GO:0005750	GO:0045275	the more general respiratory complex has been tagged and normalized, instead of the mitochondrial
20427570_13	complex III	complex III	GO:0005750	GO:0045275	the more general respiratory complex has been tagged and normalized, instead of the mitochondrial
21347277_5	RFC	Ctf18-RFC	GO:0005663	GO:0031390	the more specific complex has been tagged and normalized, due to a dictionary error
21347277_5	RFC	Ctf18-RFC	GO:0005663	GO:0031390	the more specific complex has been tagged and normalized, due to a dictionary error
21347277_5	RFC	Ctf18-RFC	GO:0005663	GO:0031390	the more specific complex has been tagged and normalized, due to a dictionary error
15223	cytochrome c oxidase	cytochrome c oxidase	-	GO:0045277	annotation error

Supplementary Table 2: Hyper-parameters used for building the Transformer-based NER system

Models	**RoBERTa-large-PM-M3-Voc** (download) (paper): used for building the Transformer-based named entity recognition system. This is a large model with 24 hidden layers in the transformer encoder.
Max sequence length	128
Learning rate	5e-05, 4e-05, 3e-05, 2e-05, 1e-05, 5e-06
Mini-batch size	2, 4, 8, 16, 32
Number of epochs	1, 2, 3, 4, 5, 6, 7, 8, 9, 10
Repetitions	3

Supplementary Table 3: Error analysis results for Jensenlab tagger run on the CoNECo test set

PubMed ID	FP/ FN	error count	entity	error type	comment
1939122	FN	1	30 S ribosomal subunits	dictionary error	synonym not in GO
1939122	FN	1	28 S	dictionary error	synonym not in GO
3453108	FN	1	high density lipoprotein	dictionary error	synonym not in GO
8407894	FN	1	TFIIIB	dictionary error	synonym not in GO
8407894	FN	3	RNA polymerase III	dictionary error	block list
9367446	FN	1	alpha(v)beta1	dictionary error	synonym not in GO
9857197	FN	1	NPC	dictionary error	block list
10617773	FN	2	collagen	dictionary error	synonym not in GO
10770935	FN	3	DRIP	dictionary error	synonym not in GO
12504026	FN	1	IkappaB	dictionary error	synonym not in GO
12815069	FN	2	TRAIL	dictionary error	synonym not in GO
15257288	FN	1	type I collagen	dictionary error	synonym not in GO
15257288	FN	5	collagen	dictionary error	synonym not in GO
15449939	FN	1	type I human interferon receptor	dictionary error	synonym not in GO
15886116	FN	1	T-cell antigen receptor	dictionary error	synonym not in GO
15886116	FN	4	TCR	dictionary error	block list
16024914	FN	1	HDL	dictionary error	block list
16717095	FN	2	cytosolic chaperonin complex	dictionary error	synonym not in GO
16717095	FN	3	CCT	dictionary error	synonym not in GO
17363904	FN	1	TCR	dictionary error	block list
17532339	FN	1	E1	dictionary error	block list
17532339	FN	3	PDC	dictionary error	synonym not in GO
17615152	FN	1	activator protein-1	dictionary error	synonym not in GO
17707230	FN	4	Pol I	dictionary error	synonym not in GO
18391171	FN	1	actomyosin	dictionary error	synonym not in GO
18838202	FN	1	nuclear factor-kappaB	dictionary error	synonym not in GO
18838202	FN	1	tumor necrosis factor (TNF)-related apoptosis-inducing ligand	dictionary error	synonym not in GO
18838202	FN	9	TRAIL	dictionary error	synonym not in GO

PubMed ID	FP/ FN	error count	entity	error type	comment
19017259	FN	1	tissue factor-activated FVII	dictionary error	synonym not in GO
19503082	FN	2	AMPARs	dictionary error	synonym not in GO
19503082	FN	4	AMPAR	dictionary error	synonym not in GO
19637179_9	FN	1	COX	dictionary error	synonym not in GO
19684574_7	FN	1	RISC	dictionary error	block list
19684574_7	FN	7	miRISC	dictionary error	synonym not in GO
19706605	FN	3	proteosome	dictionary error	synonym not in GO
20427570_13	FN	1	complexes III	dictionary error	synonym not in GO
20427570_13	FN	2	complex IV	dictionary error	synonym not in GO
20427570_13	FN	2	FoF1-ATPase	dictionary error	synonym not in GO
23810392	FN	1	nuclear factor (NF)-kappaB	dictionary error	synonym not in GO
24498436	FN	1	RNA polymerase II	dictionary error	block list
24498436	FN	2	Pol II	dictionary error	synonym not in GO
24498436	FN	2	DDR	dictionary error	term not in GO
24498436	FN	3	Pol V	dictionary error	synonym not in GO
26052893_5	FN	1	ECF transporter	dictionary error	synonym not in GO
26052893_5	FN	1	ECF transporters	dictionary error	synonym not in GO
27350684	FN	2	3'-end processing	dictionary error	synonym not in GO
28515143_17	FN	2	Mediator	dictionary error	block list
28806752_11	FN	1	IgG	dictionary error	synonym not in GO
20427570_13	FN	1	IV	discontinuous entity	
2462567	FP	7	enolase	ambiguous name	family
2981876	FP	4	p35	ambiguous name	protein
8641455	FP	2	SNARE	ambiguous name	family
8663127	FP	1	TSC	ambiguous name	other entity
8798525	FP	3	palmitoyltransferase	ambiguous name	family
8932298	FP	1	casein kinase II	ambiguous name	family
9150262	FP	3	global genome repair	ambiguous name	general term
9195882	FP	1	NADPH oxidase	ambiguous name	family
9497260	FP	1	phosphomannomutase	ambiguous name	family

PubMed ID	FP/ FN	error count	entity	error type	comment
9497260	FP	2	PMM2	ambiguous name	family
10446169	FP	1	PS1	ambiguous name	family
10446169	FP	4	presenilins	ambiguous name	family
10666337	FP	1	topoisomerase II	ambiguous name	protein
10763828	FP	1	docking protein	ambiguous name	general term
11121043	FP	1	BMP receptors	ambiguous name	general term
11121043	FP	4	Ski	ambiguous name	protein
11874917	FP	2	nonmuscle myosin	ambiguous name	family
14562105	FP	2	glucose transporter	ambiguous name	general term
14752096	FP	13	AML1	ambiguous name	protein
16055508	FP	1	Kinesin	ambiguous name	family
18391171	FP	1	capping proteins	ambiguous name	general term
20129058	FP	1	DnaB helicase	ambiguous name	protein
20129058	FP	1	DnaB hexamer	ambiguous name	protein
20129058	FP	2	prepriming complex	ambiguous name	general term
20610737	FP	1	cyclic nucleotide phosphodiesterase	ambiguous name	general term
21807881	FP	2	SNARE	ambiguous name	family
21827752	FP	1	vesicle-tethering complex	ambiguous name	general term
21900752	FP	1	ARF	ambiguous name	family
21900752	FP	1	ARF complex	ambiguous name	family
22194618	FP	2	formate dehydrogenases	ambiguous name	family
22194618	FP	2	l-Cysteine desulfurases	ambiguous name	family
23533635	FP	2	BAG6 complex	ambiguous name	protein
23533635	FP	11	BAG6	ambiguous name	protein
27751725	FP	4	DDK	ambiguous name	protein
10189366_50	FP	1	kinesin	ambiguous name	family
19415799_9	FP	7	MinD	ambiguous name	protein
19837034_29	FP	2	replisome	ambiguous name	general term
23685356_31	FP	1	POT1	ambiguous name	protein
23840694_26	FP	2	SNARE	ambiguous name	family
15492226	FP	1	TAK1 kinase complex	annotation error	

PubMed ID	FP/ FN	error count	entity	error type	comment
21807881	FP	1	COG complex	annotation error	
21807881	FP	1	conserved oligomeric Golgi	annotation error	
21807881	FP	4	COG	annotation error	
25107275	FP	1	clathrin	annotation error	
21700221_12	FP	1	APC/C	annotation error	
11809749	FP	1	polyribosomes	dictionary error	obsolete GO term
13678582	FP	1	cyclin B1-Cdk1 complex	dictionary error	name should be blocked
15492226	FP	1	Ubc13-Uev1A	dictionary error	name should be blocked
19228687	FP	1	MSH2/MSH6	dictionary error	name should be blocked
19228687	FP	1	MSH2/MSH3	dictionary error	name should be blocked
20129058	FP	1	DnaB-DnaC complex	dictionary error	name should be blocked
21807881	FP	1	Syntaxin 6-Syntaxin16-Vti1a	dictionary error	name should be blocked
22033972	FP	2	Swi5-Sfr1	dictionary error	name should be blocked
22033972	FP	6	Swi5-Sfr1 complex	dictionary error	name should be blocked
9857197	FP	2	AP-1	part-of longer entity	
11521196	FP	1	prefoldin	part-of longer entity	
16912035	FP	1	low density lipoprotein	part-of longer entity	
16912035	FP	2	LDL	part-of longer entity	
17015689	FP	1	IKK	part-of longer entity	
17015689	FP	2	IkappaB kinase	part-of longer entity	
17449811	FP	1	CALCINEURIN	part-of longer entity	
17532339	FP	1	troponin	part-of longer entity	
17532339	FP	2	pyruvate dehydrogenase	part-of longer entity	

Supplementary Table 4: Error analysis results for transformer-based tagger run on the CoNECo test set.

PubMed ID	FP/FN	error count	entity	error type	comment
27350684	FN	2	3'-end processing	ambiguous name	mislabeling; potentially mistaken for biological process
17615152	FN	1	activator protein-1	ambiguous name	mislabeling; potentially mistaken for family/protein
9367446	FN	1	alpha(v)beta1	ambiguous name	mislabeling; potentially mistaken for family/protein
19503082	FN	4	AMPAR	ambiguous name	mislabeling; potentially mistaken for family/protein
19637179	FN	1	COX	ambiguous name	mislabeling; potentially mistaken for family/protein
15223	FN	1	cytochrome c oxidase	ambiguous name	mislabeling; potentially mistaken for family/protein
26052893_5	FN	1	ECF transporter	ambiguous name	mislabeling; potentially mistaken for family/protein
26052893_5	FN	1	ECF transporters	ambiguous name	mislabeling; potentially mistaken for family/protein
16024914	FN	1	HDL	ambiguous name	mislabeling; potentially mistaken for family/protein
3453108	FN	1	high density lipoprotein	ambiguous name	mislabeling; potentially mistaken for family/protein
16912035	FN	1	LDL	ambiguous name	mislabeling; potentially mistaken for family/protein
8232104	FN	1	low density lipoprotein	ambiguous name	mislabeling; potentially mistaken for family/protein
24498436	FN	3	Pol V	ambiguous name	mislabeling; potentially mistaken for family/protein
21347277_5	FN	3	RFC	ambiguous name	mislabeling; potentially mistaken for family/protein
18838202	FN	9	TRAIL	ambiguous name	mislabeling; potentially mistaken for family/protein; the long form of the name is weird (tumor necrosis factor-related apoptosis-inducing ligand)
12815069	FN	2	TRAIL	ambiguous name	mislabeling; potentially mistaken for family/protein
18838202	FN	1	tumor necrosis factor (TNF)-related	ambiguous name	mislabeling; potentially mistaken for family/protein

PubMed ID	FP/FN	error count	entity	error type	comment
			apoptosis-inducing ligand		
15449939	FN	1	type I human interferon receptor	ambiguous name	mislabeling; potentially mistaken for family/protein
24498436	FN	2	DDR	unidentified model error	
9030599	FN	1	interleukin-5 receptor	unidentified model error	
9030599	FN	1	interleukin-6 receptor	unidentified model error	
17948059	FN	7	Paf1	unidentified model error	
30802237_18	FN	1	RENT	unidentified model error	
19017259	FN	1	tissue factor-activated FVII	unidentified model error	
1939122	FP	1	55 S initiation complexes	ambiguous name	ribosome is not a complex according to GO, but consists of complexes
1939122	FP	1	animal mitochondrial ribosomes	ambiguous name	ribosome is not a complex according to GO, but consists of complexes
29666278	FP	1	APC/CCDC20	ambiguous name	protein
8521815	FP	14	APO-1	ambiguous name	I think because it's mentioned as oligomerized at some point
12805216	FP	6	BCR	ambiguous name	protein
8932298	FP	1	casein kinase II	ambiguous name	family
29666278	FP	1	CRL4CDT2	ambiguous name	protein
17532339	FP	1	dihydrolipoyl acetyltransferase	ambiguous name	family
24768535	FP	1	DNA polymerases	ambiguous name	family
17532339	FP	1	E2	ambiguous name	family
17572495	FP	1	enhancersome	ambiguous name	general term

PubMed ID	FP/FN	error count	entity	error type	comment
9857197	FP	1	export complexes	ambiguous name	general term
22194618	FP	1	formate dehydrogenases	ambiguous name	family
16717095	FP	2	G protein betagamma	ambiguous name	group of complexes
16717095	FP	1	Gbeta	ambiguous name	protein
16717095	FP	6	Gbetagamma	ambiguous name	group of complexes
7524088	FP	1	Gi/Go	ambiguous name	protein
12036871	FP	1	glycoprotein (GP) Ib alpha	ambiguous name	protein
12036871	FP	1	glycoprotein Ib alpha	ambiguous name	protein
12036871	FP	2	GPIb alpha	ambiguous name	protein
18036997	FP	1	immunoglobulin	ambiguous name	family
8407894	FP	1	initiation transcription factor IIIB	ambiguous name	protein
20427570_13	FP	1	malate dehydrogenase	ambiguous name	family
1939122	FP	2	mitochondrial	ambiguous name	discontinuous entity
9362506_22	FP	3	NSF	ambiguous name	protein
24768535	FP	10	PCNA	ambiguous name	protein
25690888	FP	4	PCNA	ambiguous name	protein
25602519	FP	3	PCNA	ambiguous name	protein
20463880	FP	1	PCNA	ambiguous name	protein
16717095	FP	1	phosducin	ambiguous name	group of complexes
17707230	FP	1	polymerases	ambiguous name	family

PubMed ID	FP/FN	error count	entity	error type	comment
20129058	FP	3	primase	ambiguous name	family
19837034_29	FP	2	replisome	ambiguous name	general term
15117945	FP	1	ribosome	ambiguous name	ribosome is not a complex according to GO, but consists of complexes
1939122	FP	3	ribosomes	ambiguous name	ribosome is not a complex according to GO, but consists of complexes
29666278	FP	1	SCFSKP2	ambiguous name	protein
21807881	FP	1	SNARE	ambiguous name	family
8407894	FP	1	transcription factor IIIB	ambiguous name	protein
10866691	FP	1	type I casein	ambiguous name	family
10866691	FP	1	type I casein kinases	ambiguous name	family
25938661_25	FP	1	3BMP	ambiguous name	PDB ID
25938661_25	FP	1	4BQ6	ambiguous name	PDB ID
25380047_22	FP	2	AP-3	annotation error	
21700221_12	FP	1	APC/C	annotation error	
12805216	FP	1	B-cell antigen receptor	annotation error	
25602519	FP	5	CAF-I	annotation error	
25602519	FP	2	Chromatin Assembly Factor I	annotation error	
25107275	FP	1	clathrin	annotation error	
21807881	FP	5	COG	annotation error	
21807881	FP	1	conserved oligomeric Golgi	annotation error	
30802237	FP	5	Pol I	annotation error	
9115214	FP	3	T cell antigen receptor	annotation error	
9857197	FP	2	AP-1	part-of longer entity	

PubMed ID	FP/FN	error count	entity	error type	comment
17015689	FP	1	IkappaB kinase	part-of longer entity	
17015689	FP	1	IKK	part-of longer entity	

Supplementary Section 1: Using a custom ChatGPT as an annotator

To assess the possibility of automating document annotation for the task of `complex` NER, we have created 3 custom versions of ChatGPT 4.0. We have provided the prompts shown in ***Supplementary Section 1.1*** below to all versions of the chatbot. In the first version, we have provided the full annotation guidelines (with examples) as the instructions in the configuration of ChatGPT and created "CoNECo GPT - full" available through the Explore GPTs section of ChatGPT4.0 https://chat.openai.com/g/g-1uV7nfJTA-coneco-gpt-full. The instructions (***Supplementary Section 1.2***) correspond to the exact annotation guidelines provided to human annotators, with slight changes necessary to describe what was shown in markdown in the original documentation. We then provided the 25 prompts (***Supplementary Section 1.1***) one by one and within the same chat session. The terms identified as `complex` and how many of them are True Positives (TP), False Positives (FP), and False Negatives (FN) are shown in ***Supplementary Section 1.3***. We used the human annotations in this set of 25 documents as a gold standard to calculate performance metrics, applying the overlapping matching criterion. The F-score for IAA with the human annotators in this experiment is 20.5% (Precision=12.5%, Recall=58.2%). The low F-score is mainly a result of the detection of several False Positives. Comparing that to the over 90% IAA between the human annotators it is evident that this custom version of ChatGPT cannot be used for annotation purposes.

To explore if providing the full annotation guidelines is what creates confusion to this model, we decided to provide a smaller set of instructions without examples. We have created "CoNECo GPT - small", available through https://chat.openai.com/g/g-Ns0dcCn8c-coneco-gpt-small providing the set of instructions shown in ***Supplementary Section 1.4***. The prompts remain the same and detailed results of using this version are provided in ***Supplementary Section 1.5***. The F-score for IAA with the human annotators in this case is 30.5% (Precision=19.1%, Recall=75.5%). The results are better this time, but still much lower than the human annotator performance, to allow this to be a viable alternative.

Since using fewer instructions during the creation of a custom ChatGPT provided slightly better results, we decided to perform one last experiment by creating a minimal set of instructions based on our annotation guidelines. In this case, we asked the non-customized version of ChatGPT 4.0 (https://chat.openai.com) to summarize the instructions for us. We have again provided the minimal set of instructions (***Supplementary Section 1.6***) to a custom ChatGPT, thus creating "CoNECo GPT - minimal" available via https://chat.openai.com/g/g-C6Nx12aEL-coneco-gpt-minimal. The full results for the same set of 25 prompts are shown in ***Supplementary Section 1.7***. This minimal set of guidelines gives a significant boost to the IAA F-score, which now reaches 63.7%. This is mainly the result of an ~60% increase in Precision (80.6%), while the drop in Recall is around 20% (52.7%). Most false positives are cases where "CoNECo GPT - minimal" has not followed the guideline of *If entities are separated by a dash, they are examined and reported separately*} and has produced results that could easily be filtered out to increase the precision even further. Nevertheless, multiple entities are still not detected, with the recall being ~50%.

Additional experiments might be conducted to improve the recall of a custom ChatGPT, and further prompts could be provided to encourage it to identify the annotated entities. However, this falls outside the scope of evaluating ChatGPT's potential as a substitute for human annotators in either annotating or expanding the corpus. Investing more effort in refining prompts or developing more sophisticated models is arguably not going to achieve the objective of lowering the costs associated with corpus development. One more observation that is relevant for assessing the use of such a model for corpora annotation is that in contrast to human annotators, the fewer guidelines we provide to ChatGPT the better it performs. This makes it prohibitive to use a similar approach for document annotation for even more difficult tasks such as relation extraction, where it will not be possible to limit the guidelines to the degree we have done here. Finally, It should be noted that starting a new chat and providing the same set of prompts could produce different results than those presented above.

Supplementary Section 1.1: The set of prompts we provided to the custom ChatGPTs

In this work we have tested whether it is possible to use an annotation assistant for annotating documents for the CoNECo corpus, providing proper guidelines. In order to assess if this is possible we decided to use the second set of 25 documents that we used to calculate the interannotator agreement (IAA) between human annotators and provide those as prompts to custom versions of ChatGPT 4.0.

```
I want you to check the document below with ID 10359895 and I want your output to have only a comma separated list of terms that you consider protein complexes based on the rules. If you think something is a protein complex but consists of affixes (e.g. - or /) and markers, even when they are part of the same syntactic word with a Complex name, as long as there is a separating nonalphanumeric character, do not report them. And remember to check if the terms you want to report are proteins or families.:     Binding of YY1 and Oct1 to a novel element that downregulates expression of IL-5 in human T cells.  BACKGROUND: IL-5 controls development of eosinophilia and has been shown to be involved in the pathogenesis of allergic diseases. In both atopic and nonatopic asthma, elevated IL-5 has been detected in peripheral blood and the airways. IL-5 is produced mainly by activated T cells, and its expression is regulated at the transcriptional level. OBJECTIVE: This study focuses on the functional analysis of the human IL-5 (hIL-5) promoter and characterization of cis -regulatory elements and transcription factors involved in the suppression of IL-5 transcription in T cells. METHODS: Methods used in this study include DNase I footprint assays, electrophoretic mobility shift assays, and functional analysis by mammalian cell transfection involving deletion analysis and site-directed mutagenesis. RESULTS: We identified 5 protein binding regions (BRs) located within the proximal hIL-5 promoter. Functional analysis indicates that the BRs are involved in control of hIL-5 promoter activity. Two of these regions, BR3 and BR4 located at positions -102 to -73, have not previously been described as regulators of IL-5 expression in T cells. We show that the BR3 sequence contains a novel negative regulatory element located at positions -90 to -79 of the hIL-5 promoter, which binds Oct1, octamer-like, and YY1 nuclear factors. Substitution mutations, which abolished binding of these proteins to the BR3 sequence, significantly increased hIL-5 promoter activity in activated T cells. CONCLUSION: We suggest that Oct1, YY1, and octamer-like factors binding to the -90/-79 sequence within the proximal IL-5 promoter are involved in suppression of IL-5 transcription in T cells.

I want you to check the document below with ID 10660620 and I want your output to have only a comma separated list of terms that you consider protein complexes based on the rules. If you think something is a protein complex but consists of affixes (e.g. - or /) and markers, even when they are part of the same syntactic word with a Complex name, as long as there is a separating nonalphanumeric character, do not report them. And remember to check if the terms you want to report are proteins or families.:     PILRalpha, a novel immunoreceptor
```

tyrosine-based inhibitory motif-bearing protein, recruits SHP-1 upon tyrosine phosphorylation and is paired with the truncated counterpart PILRbeta. SHP-1-mediated dephosphorylation of protein tyrosine residues is central to the regulation of several cell signaling pathways, the specificity of which is dictated by the intrinsic affinity of SH2 domains for the flanking sequences of phosphotyrosine residues. By using a modified yeast two-hybrid system and SHP-1 as bait, we have cloned a human cDNA, PILRalpha, encoding a 303-amino acid immunoglobulin-like transmembrane receptor bearing two cytoplasmic tyrosines positioned within an immunoreceptor tyrosine-based inhibitory motif. Substrate trapping in combination with pervanadate treatment of 293T cells confirms that PILRalpha associates with SHP-1 in vivo upon tyrosine phosphorylation. Mutation of the tyrosine residues in PILRalpha indicates the pivotal role of the Tyr-269 residue in recruiting SHP-1. Surface plasmon resonance analysis further suggests that the association between PILRalpha-Tyr-269 and SHP-1 is mediated primarily via the amino-terminal SH2 domain of the latter. Polymerase chain reaction amplification of cDNA in combination with genomic sequence analysis revealed a second gene, PILRbeta, coding for a putative activating receptor as suggested by a truncated cytoplasmic tail and a charged lysine residue in its transmembrane region. The PILRalpha and PILRbeta genes are localized to chromosome 7 which is in contrast with the mapping of known members of the inhibitory receptor superfamily.

I want you to check the document below with ID 11331609 and I want your output to have only a comma separated list of terms that you consider protein complexes based on the rules. If you think something is a protein complex but consists of affixes (e.g. - or /) and markers, even when they are part of the same syntactic word with a Complex name, as long as there is a separating nonalphanumeric character, do not report them. And remember to check if the terms you want to report are proteins or families.: Sharp, an inducible cofactor that integrates nuclear receptor repression and activation. A yeast two-hybrid screen using the conserved carboxyl terminus of the nuclear receptor corepressor SMRT as a bait led to the isolation of a novel human gene termed SHARP (SMRT/HDAC1 Associated Repressor Protein). SHARP is a potent transcriptional repressor whose repression domain (RD) interacts directly with SMRT and at least five members of the NuRD complex including HDAC1 and HDAC2. In addition, SHARP binds to the steroid receptor RNA coactivator SRA via an intrinsic RNA binding domain and suppresses SRA-potentiated steroid receptor transcription activity. Accordingly, SHARP has the capacity to modulate both liganded and nonliganded nuclear receptors. Surprisingly, the expression of SHARP is itself steroid inducible, suggesting a simple feedback mechanism for attenuation of the hormonal response.

I want you to check the document below with ID 11359852 and I want your output to have only a comma separated list of terms that you consider protein complexes based on the rules. If you think something is a protein complex but consists of affixes (e.g. - or /) and markers, even when they are part of the same syntactic word with a Complex name, as long as there is a separating nonalphanumeric character, do not report them. And remember to check if the terms you want to report are proteins or families.: Thiopalmitoylation of myelin proteolipid protein epitopes enhances immunogenicity and encephalitogenicity. Proteolipid protein (PLP) is the most abundant protein of CNS myelin, and is posttranslationally acylated by covalent attachment of long chain fatty acids to cysteine residues via a thioester linkage. Two of the acylation sites are within epitopes of PLP that are encephalitogenic in SJL/J mice (PLP(104-117) and PLP(139-151)) and against which increased immune responses have been detected in some multiple sclerosis patients. It is known that attachment of certain types of lipid side chains to peptides can result in their enhanced immunogenicity. The aim of this study was to determine whether thioacylated PLP peptides, as occur in the native protein, are more immunogenic than their nonacylated counterparts, and whether thioacylation influences the development of autoreactivity and experimental autoimmune encephalomyelitis. The results show that in comparison with nonacylated peptides, thioacylated PLP lipopeptides can induce greater T cell and Ab responses to both the acylated and nonacylated peptides. They also enhanced the development and chronicity of experimental autoimmune encephalomyelitis. Synthetic peptides in which the fatty acid was attached via an amide linkage at the N terminus were not encephalitogenic, and they induced greater proportions of CD8+ cells in initial in vitro stimulation. Therefore, the lability and the site of the linkage between the peptide and fatty acid may be important for induction of encephalitogenic CD4+ T cells. These results suggest that immune responses induced by endogenous thioacylated lipopeptides may contribute to the immunopathogenesis of chronic experimental demyelinating diseases and multiple sclerosis.

I want you to check the document below with ID 11675392 and I want your output to have only a comma separated list of terms that you consider protein complexes based on the rules. If you think something is a protein complex but consists of affixes (e.g. - or /) and markers, even when they are part of the same syntactic word with a Complex name, as long as there is

a separating nonalphanumeric character, do not report them. And remember to check if the terms you want to report are proteins or families.: Phosphoisoprenoids modulate association of Rab geranylgeranyltransferase with REP-1. Rab geranylgeranyltransferase (RabGGTase or GGTase-II) catalyzes the post-translational prenylation of Rab proteins. Rab proteins are recognized as substrates only when they are complexed to Rab Escort Protein (REP). The classical model of prenylation complex assembly assumes initial formation of the Rab.REP binary complex, which subsequently binds to RabGGTase loaded with the isoprenoid donor geranylgeranyl pyrophosphate (GGpp). We demonstrate here that REP-1 can also associate with RabGGTase in the absence of Rab protein and that this interaction is dramatically strengthened by the presence of phosphoisoprenoids such as GGpp. The GGpp-dependent interaction between RabGGTase and REP-1 was observed using affinity precipitations and gel filtration and was quantitated on the basis of fluorescence assays. In the presence of GGpp, REP-1 binds to RabGGTase with a K(d) value of approximately 10 nm, while in its absence the affinity between the two proteins is in the micromolar range. We further demonstrate that binding of Rab7 to the RabGGTase.GGpp.REP-1 complex occurs without prior dissociation of REP-1. Analysis of binding and prenylation rate constants indicate that the RabGGTase.GGpp.REP-1 complex can function as a kinetically competent intermediate of the prenylation reaction. We conclude that, depending on the prevailing concentrations, binding of REP-1 to RabGGTase in the presence of GGpp may serve as an alternative pathway for the assembly of the prenylation machinery in vivo. Implications of these findings for the role of REP-1 in the prenylation reaction are discussed.

I want you to check the document below with ID 11832247 and I want your output to have only a comma separated list of terms that you consider protein complexes based on the rules. If you think something is a protein complex but consists of affixes (e.g. - or /) and markers, even when they are part of the same syntactic word with a Complex name, as long as there is a separating nonalphanumeric character, do not report them. And remember to check if the terms you want to report are proteins or families.: Direct interaction between Rab3b and the polymeric immunoglobulin receptor controls ligand-stimulated transcytosis in epithelial cells. We have examined the role of rab3b in epithelial cells. In MDCK cells, rab3b localizes to vesicular structures containing the polymeric immunoglobulin receptor (pIgR) and located subjacent to the apical surface. We found that GTP-bound rab3b directly interacts with the cytoplasmic domain of pIgR. Binding of dIgA to pIgR causes a dissociation of the interaction with rab3b, a process that requires dIgA-mediated signaling, Arg657 in the cytoplasmic domain of pIgR, and possibly GTP hydrolysis by rab3b. Binding of dIgA to pIgR at the basolateral surface stimulates subsequent transcytosis to the apical surface. Overexpression of GTP-locked rab3b inhibits dIgA-stimulated transcytosis. Together, our data demonstrate that a rab protein can bind directly to a specific cargo protein and thereby control its trafficking.

I want you to check the document below with ID 11917125 and I want your output to have only a comma separated list of terms that you consider protein complexes based on the rules. If you think something is a protein complex but consists of affixes (e.g. - or /) and markers, even when they are part of the same syntactic word with a Complex name, as long as there is a separating nonalphanumeric character, do not report them. And remember to check if the terms you want to report are proteins or families.: Human receptors for sweet and umami taste. The three members of the T1R class of taste-specific G protein-coupled receptors have been hypothesized to function in combination as heterodimeric sweet taste receptors. Here we show that human T1R2/T1R3 recognizes diverse natural and synthetic sweeteners. In contrast, human T1R1/T1R3 responds to the umami taste stimulus l-glutamate, and this response is enhanced by 5'-ribonucleotides, a hallmark of umami taste. The ligand specificities of rat T1R2/T1R3 and T1R1/T1R3 correspond to those of their human counterparts. These findings implicate the T1Rs in umami taste and suggest that sweet and umami taste receptors share a common subunit.

I want you to check the document below with ID 12022229 and I want your output to have only a comma separated list of terms that you consider protein complexes based on the rules. If you think something is a protein complex but consists of affixes (e.g. - or /) and markers, even when they are part of the same syntactic word with a Complex name, as long as there is a separating nonalphanumeric character, do not report them. And remember to check if the terms you want to report are proteins or families.: Yeast Pescadillo is required for multiple activities during 60S ribosomal subunit synthesis. The Pescadillo protein was identified via a developmental defect and implicated in cell cycle progression. Here we report that human Pescadillo and its yeast homolog (Yph1p or Nop7p) are localized to the nucleolus. Depletion of Nop7p leads to nuclear accumulation of pre-60S particles, indicating a defect in subunit export, and it interacts genetically with a tagged form of the ribosomal protein Rpl25p, consistent with a role in subunit assembly. Two pre-rRNA

processing pathways generate alternative forms of the 5.8S rRNA, designated 5.8S(L) and 5.8Ss. In cells depleted for Nop7p, the 27SA3 pre-rRNA accumulated, whereas later processing intermediates and the mature 5.8Ss rRNA were depleted. Less depletion was seen for the 5.8S(L) pathway. TAP-tagged Nop7p coprecipitated precursors to both 5.8S(L) and 5.8Ss but not the mature rRNAs. We conclude that Nop7p is required for efficient exonucleolytic processing of the 27SA3 pre-rRNA and has additional functions in 60S subunit assembly and transport. Nop7p is a component of at least three different pre-60S particles, and we propose that it carries out distinct functions in each of these complexes.

I want you to check the document below with ID 14592967 and I want your output to have only a comma separated list of terms that you consider protein complexes based on the rules. If you think something is a protein complex but consists of affixes (e.g. - or /) and markers, even when they are part of the same syntactic word with a Complex name, as long as there is a separating nonalphanumeric character, do not report them. And remember to check if the terms you want to report are proteins or families.: Cytosolic HSP90 associates with and modulates the Arabidopsis RPM1 disease resistance protein. The Arabidopsis protein RPM1 activates disease resistance in response to Pseudomonas syringae proteins targeted to the inside of the host cell via the bacterial type III delivery system. We demonstrate that specific mutations in the ATP-binding domain of a single Arabidopsis cytosolic HSP90 isoform compromise RPM1 function. These mutations do not affect the function of related disease resistance proteins. RPM1 associates with HSP90 in plant cells. The Arabidopsis proteins RAR1 and SGT1 are required for the action of many R proteins, and display some structural similarity to HSP90 co-chaperones. Each associates with HSP90 in plant cells. Our data suggest that (i) RPM1 is an HSP90 client protein; and (ii) RAR1 and SGT1 may function independently as HSP90 cofactors. Dynamic interactions among these proteins can regulate RPM1 stability and function, perhaps similarly to the formation and regulation of animal steroid receptor complexes.

I want you to check the document below with ID 15220471 and I want your output to have only a comma separated list of terms that you consider protein complexes based on the rules. If you think something is a protein complex but consists of affixes (e.g. - or /) and markers, even when they are part of the same syntactic word with a Complex name, as long as there is a separating nonalphanumeric character, do not report them. And remember to check if the terms you want to report are proteins or families.: Silent information regulator 2 potentiates Foxo1-mediated transcription through its deacetylase activity. Longevity regulatory genes include the Forkhead transcription factor FOXO and the NAD-dependent histone deacetylase silent information regulator 2 (Sir2). Genetic studies demonstrate that Sir2 acts to extend lifespan in Caenorhabditis elegans upstream of DAF-16, a member of the FOXO family, in the insulin-like signaling pathway. However, the molecular mechanisms underlying the requirement of DAF-16 activity in Sir2-mediated longevity remain unknown. Here we show that reversible acetylation of Foxo1 (also known as FKHR), the mouse DAF-16 ortholog, modulates its transactivation function. cAMP-response element-binding protein (CREB)-binding protein binds and acetylates Foxo1 at the K242, K245, and K262 residues, the modification of which is involved in the attenuation of Foxo1 as a transcription factor. Conversely, Sir2 binds and deacetylates Foxo1 at residues acetylated by cAMP-response element-binding protein-binding protein. Sir2 is recruited to insulin response sequence-containing promoter and increases the expression of manganese superoxide dismutase and p27(kip1) in a deacetylase-activity-dependent manner. Our findings establish Foxo1 as a direct and functional target for Sir2 in mammalian systems.

I want you to check the document below with ID 16274718 and I want your output to have only a comma separated list of terms that you consider protein complexes based on the rules. If you think something is a protein complex but consists of affixes (e.g. - or /) and markers, even when they are part of the same syntactic word with a Complex name, as long as there is a separating nonalphanumeric character, do not report them. And remember to check if the terms you want to report are proteins or families.: S-palmitoylation modulates estrogen receptor alpha localization and functions. 17beta-Estradiol (E2) acts as a chemical messenger in target tissues inducing both slow nuclear and rapid extra-nuclear responses. E2 binds to its cognate nuclear receptors (ER) resulting in the activation of target gene transcription in the nucleus. In addition to these genomic effects, E2 modulates cell functions through rapid non-genomic actions. Stimulation of G-proteins, Ca(2+) influx, inositol phosphate generation as well as phospholipase C, ERK/MAPK, and PI3K/AKT activation all occur within seconds to minutes after E2 binding to a small population of ERalpha located at the plasma membrane. The great impact of these rapid signals on cell physiology renders central the knowledge of the structural bases and mechanisms that mediate extra-nuclear signaling by E2. Several laboratories, including our own, have recently elucidated the structural requirements for localization and function of plasma membrane

ERalpha. This review summarizes the molecular mechanisms of E2-induced rapid non-genomic actions relevant for cell functions, highlighting the role of lipid modification (i.e., palmitoylation) in the ERalpha localization to and residence at the plasma membrane.

I want you to check the document below with ID 17911105 and I want your output to have only a comma separated list of terms that you consider protein complexes based on the rules. If you think something is a protein complex but consists of affixes (e.g. - or /) and markers, even when they are part of the same syntactic word with a Complex name, as long as there is a separating nonalphanumeric character, do not report them. And remember to check if the terms you want to report are proteins or families.: Active gamma-secretase complexes contain only one of each component. Gamma-secretase is an intramembrane aspartyl protease complex that cleaves type I integral membrane proteins, including the amyloid beta-protein precursor and the Notch receptor, and is composed of presenilin, Pen-2, nicastrin, and Aph-1. Although all four of these membrane proteins are essential for assembly and activity, the stoichiometry of the complex is unknown, with the number of presenilin molecules present being especially controversial. Here we analyze functional gamma-secretase complexes, isolated by immunoprecipitation from solubilized membrane fractions and able to produce amyloid beta-peptides and amyloid beta-protein precursor intracellular domain. We show that the active isolated protease contains only one presenilin per complex, which excludes certain models of the active site that require aspartate dyads formed between two presenilin molecules. We also quantified components in the isolated complexes by Western blot using protein standards and found that the amounts of Pen-2 and nicastrin were the same as that of presenilin. Moreover, we found that one Aph-1 was not co-immunoprecipitated with another in active complexes, evidence that Aph-1 is likewise present as a monomer. Taken together, these results demonstrate that the stoichiometry of gamma-components presenilin:Pen-2:nicastrin:Aph-1 is 1:1:1:1.

I want you to check the document below with ID 18253500 and I want your output to have only a comma separated list of terms that you consider protein complexes based on the rules. If you think something is a protein complex but consists of affixes (e.g. - or /) and markers, even when they are part of the same syntactic word with a Complex name, as long as there is a separating nonalphanumeric character, do not report them. And remember to check if the terms you want to report are proteins or families.: Fine mapping of posttranslational modifications of the linker histone H1 from Drosophila melanogaster. The linker histone H1 binds to the DNA in between adjacent nucleosomes and contributes to chromatin organization and transcriptional control. It is known that H1 carries diverse posttranslational modifications (PTMs), including phosphorylation, lysine methylation and ADP-ribosylation. Their biological functions, however, remain largely unclear. This is in part due to the fact that most of the studies have been performed in organisms that have several H1 variants, which complicates the analyses. We have chosen Drosophila melanogaster, a model organism, which has a single H1 variant, to approach the study of the role of H1 PTMs during embryonic development. Mass spectrometry mapping of the entire sequence of the protein showed phosphorylation only in the ten N-terminal amino acids, mostly at S10. For the first time, changes in the PTMs of a linker H1 during the development of a multicellular organism are reported. The abundance of H1 monophosphorylated at S10 decreases as the embryos age, which suggests that this PTM is related to cell cycle progression and/or cell differentiation. Additionally, we have found a polymorphism in the protein sequence that can be mistaken with lysine methylation if the analysis is not rigorous.

I want you to check the document below with ID 18342605 and I want your output to have only a comma separated list of terms that you consider protein complexes based on the rules. If you think something is a protein complex but consists of affixes (e.g. - or /) and markers, even when they are part of the same syntactic word with a Complex name, as long as there is a separating nonalphanumeric character, do not report them. And remember to check if the terms you want to report are proteins or families.: The Glc7 phosphatase subunit of the cleavage and polyadenylation factor is essential for transcription termination on snoRNA genes. Glc7, the yeast protein phosphatase 1, is a component of the cleavage and polyadenylation factor (CPF). Here we show that downregulation of Glc7, or its dissociation from CPF in the absence of CPF subunits Ref2 or Swd2, results in similar snoRNA termination defects. Overexpressing a C-terminal fragment of Sen1, a superfamily I helicase required for snoRNA termination, suppresses the growth and termination defects associated with loss of Swd2 or Ref2, but not Glc7. Suppression by Sen1 requires nuclear localization and direct interaction with Glc7, which can dephosphorylate Sen1 in vitro. The suppressing fragment, and in a similar manner full-length Sen1, copurifies with the snoRNA termination factors Nrd1 and Nab3, suggesting loss of Glc7 from CPF can be compensated by recruiting Glc7 to

Nrd1-Nab3 through Sen1. Swd2 is also a subunit of the Set1c histone H3K4 methyltransferase complex and is required for its stability and optimal methyltransferase activity.

I want you to check the document below with ID 19608646 and I want your output to have only a comma separated list of terms that you consider protein complexes based on the rules. If you think something is a protein complex but consists of affixes (e.g. - or /) and markers, even when they are part of the same syntactic word with a Complex name, as long as there is a separating nonalphanumeric character, do not report them. And remember to check if the terms you want to report are proteins or families.: The trafficking/interaction of eNOS and caveolin-1 induced by insulin modulates endothelial nitric oxide production. Endothelial nitric oxide synthase (eNOS) activity is tightly regulated by posttranscriptional modification and its subcellular localization. Here we examined whether insulin modulates nitric oxide (NO) production by regulating eNOS subcellular localization. We used confocal microscopy and immunoblots to examine the time course for 1) subcellular targeting/association of eNOS and caveolin-1 (CAV-1); 2) eNOS Ser(1179) phosphorylation; and 3) NO production in cultured bovine aorta endothelial cells. Serum starvation increased eNOS/CAV-1 localization to the perinuclear region. Adding insulin provoked their prompt translocation to and association at the plasma membrane (PM). Specific monoclonal antibodies against either CAV-1 or eNOS coimmunoprecipitated the other from bovine aorta endothelial cell membrane extracts, and insulin increased this interaction. Insulin stimulated NO production transiently despite a persistent eNOS Ser(1179) phosphorylation. The decline of NO production correlated temporally to insulin-induced translocation of eNOS and CAV-1 to PM. Knockdown of CAV-1 expression with a specific small interfering RNA duplex resulted in eNOS redistributing to the perinuclear region and nearly doubled insulin-induced NO production. Inhibition of phosphatidylinositol 3-kinase activity with wortmannin not only significantly inhibited insulin-induced translocation of eNOS and CAV-1 to PM but also blocked insulin-induced interaction of CAV-1 with eNOS at PM. Insulin increased incorporation of [(3)H]palmitic acid into eNOS immunoprecipitates by approximately 140%. Insulin-induced translocation of eNOS and CAV-1 to PM was palmitoylation dependent. Inhibiting eNOS and CAV-1 palmitoylation enhanced the NO production while blocking the translocation of eNOS and CAV-1 to PM induced by insulin. These data show that insulin acutely regulates eNOS and CAV-1 trafficking to PM of vascular endothelial cells where their interaction can regulate eNOS activity.

I want you to check the document below with ID 21884581_16 and I want your output to have only a comma separated list of terms that you consider protein complexes based on the rules. If you think something is a protein complex but consists of affixes (e.g. - or /) and markers, even when they are part of the same syntactic word with a Complex name, as long as there is a separating nonalphanumeric character, do not report them. And remember to check if the terms you want to report are proteins or families.: At 24 h before transfection, cells (2 x 105-aliquots) were plated in 6-well plates and transfected with increasing amounts of B7.1-YPet-, B7-H1-YPet- or IR-YPet-expressing plasmids (10 to 500 ng/well), and constant amounts (10 ng-aliquots) of plasmid expressing B7-H1-Rluc8 or B7.1-Rluc8 fusion protein. 48 h later, cells were collected and washed twice with PBS, and aliquots were placed in 384-well plates. Coelenterazine H substrate was added at a final molarity of 5 muM, and BRET was measured immediately. To analyze the effect of the penton, cells were incubated for 5 min without or with penton protein solution at 0.33, 0.66 or 132 ng/muL, followed by Coelenterazine H addition and BRET measurement. BRET was monitored using a lumino/fluorometer (Mithras; Berthold Technologies, France), allowing for the sequential integration of luminescence with two filter settings (Rluc filter, 485 +/- 10 nm; YFP filter, 530 +/- 12.5 nm). The emission signal values obtained at 530 nm were divided by the emission signal values obtained at 485 nm. The BRET ratio was calculated by dividing the emission signal value obtained with coexpressed donor and acceptor by that obtained with the donor protein expressed alone. Data from at least three independent experiments were averaged, and results expressed as milliBRET (mBRET), corresponding to the BRET ratio multiplied by 1,000. Donor saturation curves were determined as previously described.

I want you to check the document below with ID 22723847_14 and I want your output to have only a comma separated list of terms that you consider protein complexes based on the rules. If you think something is a protein complex but consists of affixes (e.g. - or /) and markers, even when they are part of the same syntactic word with a Complex name, as long as there is a separating nonalphanumeric character, do not report them. And remember to check if the terms you want to report are proteins or families.: Given the role of Ubp9, Ubp13 and Duf1 for normal mitochondria function, we first checked the localization of these three proteins. We found that these three proteins had similar distributions: GFP-tagged proteins were found mostly in the cytoplasm, in various growth conditions (data

not shown), as described in databases for Ubp9 and Duf1. Biochemical fractionation of chromosomal-encoded HA-tagged proteins indicated that, in addition to the cytoplasmic soluble fraction, these proteins also display a membrane-bound fraction, possibly associated with mitochondria (Supplemental Fig. S2 B-C).

I want you to check the document below with ID 23028367 and I want your output to have only a comma separated list of terms that you consider protein complexes based on the rules. If you think something is a protein complex but consists of affixes (e.g. - or /) and markers, even when they are part of the same syntactic word with a Complex name, as long as there is a separating nonalphanumeric character, do not report them. And remember to check if the terms you want to report are proteins or families.: Rif2 promotes a telomere fold-back structure through Rpd3L recruitment in budding yeast. Using a genome-wide screening approach, we have established the genetic requirements for proper telomere structure in Saccharomyces cerevisiae. We uncovered 112 genes, many of which have not previously been implicated in telomere function, that are required to form a fold-back structure at chromosome ends. Among other biological processes, lysine deacetylation, through the Rpd3L, Rpd3S, and Hda1 complexes, emerged as being a critical regulator of telomere structure. The telomeric-bound protein, Rif2, was also found to promote a telomere fold-back through the recruitment of Rpd3L to telomeres. In the absence of Rpd3 function, telomeres have an increased susceptibility to nucleolytic degradation, telomere loss, and the initiation of premature senescence, suggesting that an Rpd3-mediated structure may have protective functions. Together these data reveal that multiple genetic pathways may directly or indirectly impinge on telomere structure, thus broadening the potential targets available to manipulate telomere function.

I want you to check the document below with ID 23427262_17 and I want your output to have only a comma separated list of terms that you consider protein complexes based on the rules. If you think something is a protein complex but consists of affixes (e.g. - or /) and markers, even when they are part of the same syntactic word with a Complex name, as long as there is a separating nonalphanumeric character, do not report them. And remember to check if the terms you want to report are proteins or families.: Our previous study showed that the major role of the loop lies in the recruitment of the microtubule-associated protein Dis1, a founding member of the TOG/XMAP215 microtubule-associated protein family, and in the ndc80-21 mutant (L405P), Dis1 delocalized from the mitotic kinetochore. Accordingly, we examined Dis1 localization in the ndc80-NH12 mutant and quantified its intensity. Intriguingly, the signal intensities of Dis1 at the mitotic kinetochore were indistinguishable from those of wild-type cells (Figure 4A).

I want you to check the document below with ID 26320581_12 and I want your output to have only a comma separated list of terms that you consider protein complexes based on the rules. If you think something is a protein complex but consists of affixes (e.g. - or /) and markers, even when they are part of the same syntactic word with a Complex name, as long as there is a separating nonalphanumeric character, do not report them. And remember to check if the terms you want to report are proteins or families.: The catalytic domain of MLL4(tag) adopts the canonical beta-fold structure observed for other SET domains, and closely resembles that of MLL1 (Figures 3C and 3D). The N-flanking region of MLL4 forms an extended helical structure, and is markedly different from the extended loop observed for MLL1. Conserved features include the cofactor binding site, which is located in a surface pocket created by the intersection of the SET-N, SET-C, and postSET domains. The position of the substrate binding channel is located between the SET-I and postSET domains, and is indicated in Figure 3C. The C-terminal tag immediately follows the conserved Cys4Zn cluster and enters the substrate binding channel before turning and passing over the cofactor. The tag was required for crystallization but resulted in a catalytically dead construct (Figure 3B), and its impact on the MLL4 SET domain structure and activity is discussed in detail in Figure S3. An equivalent MLL4 construct prepared with an N-terminal tag, like the MLL4(WIN) construct, had high intrinsic activity (Figure 3B), indicating that the determinants of this intrinsic activity are contained within this region of the SET domain. The lack of methyltransferase activity observed with the C-terminally tagged construct can be attributed to partial blocking of the substrate binding channel and interactions with the cofactor.

I want you to check the document below with ID 7667268 and I want your output to have only a comma separated list of terms that you consider protein complexes based on the rules. If you think something is a protein complex but consists of affixes (e.g. - or /) and markers, even when they are part of the same syntactic word with a Complex name, as long as there is a separating nonalphanumeric character, do not report them. And remember to check if the terms you want to report are proteins or families.: Evolutionary conservation of human

TATA-binding-polypeptide-associated factors TAFII31 and TAFII80 and interactions of TAFII80 with other TAFs and with general transcription factors. Human transcription initiation factor TFIID is composed of the TATA-binding polypeptide (TBP) and at least 13 TBP-associated factors (TAFs) that collectively or individually are involved in activator-dependent transcription. To investigate protein-protein interactions involved in TFIID assembly and in TAF-mediated activator functions, we have cloned and expressed cDNAs encoding human TAFII80 and TAFII31. Coimmunoprecipitation assays showed that TAFII80 interacted with TAFII250, TAFII31, TAFII20, and TBP, but not with TAFII55. Similar assays showed that TAFII80 interacted with TFIIE alpha and with TFIIF alpha (RAP74) but not with TFIIB, TFIIE beta, or TFIIF beta (RAP30). Further studies with TAFII80 mutations revealed three distinct interaction domains which fall within regions conserved in human TAFII80, Drosophila TAFII60, and yeast TAFII60. The N terminus of TAFII80 (residues 1-100) interacts with both TAFII31 and TAFII20, while two C-terminal regions are involved, respectively, in interactions with TAFII250 and TFIIF alpha (RAP74) (residues 203-276) and with TBP and TFIIE alpha (residues 377-505). The interactions between TAFII80 and general factors TFIIE alpha and TFIIF alpha (RAP74) could be important for recruitment of GTFs during activator-dependent transcription. Because TAFs 80, 31, and 20 show sequence similarities to histones H4, H3, and H2B, as well as some parallel interactions, this subset of TAFs may form a related core structure within TFIID.

I want you to check the document below with ID 7957109 and I want your output to have only a comma separated list of terms that you consider protein complexes based on the rules. If you think something is a protein complex but consists of affixes (e.g. - or /) and markers, even when they are part of the same syntactic word with a Complex name, as long as there is a separating nonalphanumeric character, do not report them. And remember to check if the terms you want to report are proteins or families.:   A proteasome inhibitor prevents activation of NF-kappa B and stabilizes a newly phosphorylated form of I kappa B-alpha that is still bound to NF-kappa B.   Activation of the inducible transcription factor NF-kappa B involves removal of the inhibitory subunit I kappa B-alpha from a latent cytoplasmic complex. It has been reported that I kappa B-alpha is subject to both phosphorylation and proteolysis in the process of NF-kappa B activation. In this study, we present evidence that the multicatalytic cytosolic protease (proteasome) is involved in the degradation of I kappa B-alpha. Micromolar amounts of the peptide Cbz-Ile-Glu(O-t-Bu)-Ala-leucinal (PSI), a specific inhibitor of the chymotrypsin-like activity of the proteasome, prevented activation of NF-kappa B in response to tumor necrosis factor-alpha (TNF) and okadaic acid (OA) through inhibition of I kappa B-alpha degradation. The m-calpain inhibitor Cbz-Leu-leucinal was ineffective. In the presence of PSI, a newly phosphorylated form of I kappa B-alpha accumulated in TNF- and OA-stimulated cells. However, the covalent modification of I kappa B-alpha was not sufficient for activation of NF-kappa B: no substantial NF-kappa B DNA binding activity appeared in cells because the newly phosphorylated form of I kappa B-alpha was still tightly bound to p65 NF-kappa B. Pyrrolidinedithiocarbamate, an antioxidant inhibitor of NF-kappa B activation which did not interfere with proteasome activities, prevented de novo phosphorylation of I kappa B-alpha as well as its subsequent degradation. This suggests that phosphorylation of I kappa B-alpha is equally necessary for the activation of NF-kappa B.(ABSTRACT TRUNCATED AT 250 WORDS)

I want you to check the document below with ID 8506307 and I want your output to have only a comma separated list of terms that you consider protein complexes based on the rules. If you think something is a protein complex but consists of affixes (e.g. - or /) and markers, even when they are part of the same syntactic word with a Complex name, as long as there is a separating nonalphanumeric character, do not report them. And remember to check if the terms you want to report are proteins or families.:   In vitro and in vivo functional characterization of bovine vitamin K-dependent gamma-carboxylase expressed in Chinese hamster ovary cells. Coagulation factor IX is a serine protease for which high-level expression of biologically active protein in heterologous cells is limited due to inefficient proteolytic removal of the propeptide as well as vitamin K-dependent carboxylation of multiple amino-terminal glutamic acid residues. We have overexpressed the vitamin K-dependent gamma-carboxylase cDNA and monitored its ability to improve factor IX processing in Chinese hamster ovary (CHO) cells. From amino acid sequence analysis of bovine liver vitamin K-dependent gamma-carboxylase, degenerate oligonucleotides were used to isolate a 3.5-kbp bovine cDNA that encoded a 758-residue open reading frame. Expression of the cDNA in COS-1 and CHO cells yielded 17- and 16-fold increases in the in vitro gamma-carboxylase activity of microsomal preparations, respectively. Anti-serum raised against a predicted peptide sequence reacted with a 94-kDa polypeptide in the partially purified bovine liver preparation as well as in stably transfected CHO cells. The amount of antibody reactivity correlated with the increased ability to carboxylate a peptide

substrate in vitro. These results strongly support the conclusion that the cDNA encodes the vitamin K-dependent gamma-carboxylase. Transient transfection of the gamma-carboxylase expression vector into factor IX-expressing CHO cells did not improve the specific procoagulant activity of secreted factor IX. In contrast, transfection of an expression vector encoding the propeptide processing enzyme PACE (paired basic amino acid cleaving enzyme) did improve the specific activity of secreted factor IX by 3-fold. These results demonstrate that the ability of CHO cells to modify glutamic acid residues to gamma-carboxyglutamic acid in secreted factor IX is not limited by the expression of the vitamin K-dependent gamma-carboxylase alone.

I want you to check the document below with ID 9616112 and I want your output to have only a comma separated list of terms that you consider protein complexes based on the rules. If you think something is a protein complex but consists of affixes (e.g. - or /) and markers, even when they are part of the same syntactic word with a Complex name, as long as there is a separating nonalphanumeric character, do not report them. And remember to check if the terms you want to report are proteins or families.: Role of the CLOCK protein in the mammalian circadian mechanism. The mouse Clock gene encodes a bHLH-PAS protein that regulates circadian rhythms and is related to transcription factors that act as heterodimers. Potential partners of CLOCK were isolated in a two-hybrid screen, and one, BMAL1, was coexpressed with CLOCK and PER1 at known circadian clock sites in brain and retina. CLOCK-BMAL1 heterodimers activated transcription from E-box elements, a type of transcription factor-binding site, found adjacent to the mouse per1 gene and from an identical E-box known to be important for per gene expression in Drosophila. Mutant CLOCK from the dominant-negative Clock allele and BMAL1 formed heterodimers that bound DNA but failed to activate transcription. Thus, CLOCK-BMAL1 heterodimers appear to drive the positive component of per transcriptional oscillations, which are thought to underlie circadian rhythmicity.

I want you to check the document below with ID 9659924 and I want your output to have only a comma separated list of terms that you consider protein complexes based on the rules. If you think something is a protein complex but consists of affixes (e.g. - or /) and markers, even when they are part of the same syntactic word with a Complex name, as long as there is a separating nonalphanumeric character, do not report them. And remember to check if the terms you want to report are proteins or families.: Recruitment of CBP/p300 by the IFN beta enhanceosome is required for synergistic activation of transcription. Transcriptional activation of the IFN beta gene in response to virus infection requires the assembly of an enhanceosome, consisting of the transcriptional activators NF-kappa B, IRF1, ATF2/c-Jun, and the architectural protein HMG I(Y). The level of transcription generated by all of these activators is greater than the sum of the levels generated by individual factors, a phenomenon designated transcriptional synergy. We demonstrate that this synergy, in the context of the enhanceosome, requires a new protein-protein interaction domain in the p65 subunit of NF-kappa B. Transcriptional synergy requires recruitment of the CBP/p300 coactivator to the enhanceosome, via a new activating surface assembled from the novel p65 domain and the activation domains of all of the activators. Deletion, substitution, or rearrangement of any one of the activation domains in the context of the enhanceosome decreases both recruitment of CBP and transcriptional synergy.

Supplementary Section 1.2: CoNECO GPT full - Instructions

The “CoNECo GPT - full” available through https://chat.openai.com/g/g-1uV7nfJTA-coneco-gpt-full is a custom ChatGPT that was generated with the following set of instructions corresponding to the annotation guidelines provided to human annotators. There is a limit of 8000 characters for instructions and for that reason the GO term names were removed from the list in the bottom of the instructions:

```
You are a helpful assistant. Your task is to identify protein-containing complexes in the CoNECo corpus using the following annotation guidelines:
- Entities that fall within the scope of the Gene Ontology term protein-containing complex are the target of named entity annotations for CoNECo. Excerpt taken from Gene Ontology: A protein complex in this context is meant as a stable set of interacting proteins which can be co-purified by an acceptable method, and where the complex has been shown to exist as an isolated, functional unit in vivo. Acceptable experimental methods include stringent protein purification followed by detection of protein interaction. The following methods should be considered non-acceptable: simple immunoprecipitation, pull-down experiments from cell extracts without further purification, colocalization and 2-hybrid screening. Interactions that should not be captured as protein complexes include: 1) enzyme/substrate, receptor/ligand or any similar transient interactions, unless these are a critical part of the complex assembly or are required e.g. for the receptor to be functional; 2) proteins associated in a pull-down/co-immunoprecipitation assay with no functional link or any evidence that this is a defined biological entity rather than a loose-affinity complex; 3) any complex where the only evidence is based on genetic interaction data; 4) partial complexes, where some subunits (e.g. transmembrane ones) cannot be expressed as recombinant proteins and are excluded from experiments (in this case, independent evidence is necessary to find out the composition of the full complex, if known). Interactions that may be captured as protein complexes include: 1) enzyme/substrate or receptor/ligand if the complex can only assemble and become functional in the presence of both classes of subunits; 2) complexes where one of the members has not been shown to be physically linked to the other(s), but is a homologue of, and has the same functionality as, a protein that has been experimentally demonstrated to form a complex with the other member(s); 3) complexes whose existence is accepted based on localization and pharmacological studies, but for which experimental evidence is not yet available for the complex as a whole.
- If a term is found in Gene ontology but it is NOT a protein-containing complex, then it will NOT be considered a Complex in this effort (e.g. ribosome).
- Gene Ontology-Cellular Component (GO-CC) and specifically GO terms under protein-containing complex will be the target for Named Entity Normalization.
- If a protein-containing complex can be identified as such by the annotators, but there is no entry in Gene Ontology corresponding to the entity then it will receive an annotation as a named entity (NE), but will not have a normalization to GO-CC.
- The first resource that is trusted to resolve issues is Gene Ontology. If there is still not enough information there, inconsistencies will be resolved using Complex Portal, Reactome, CORUM, and as a last result the literature.
- For each mention of a protein-containing complex (Complex hereafter) name, the annotation aims to mark the minimal span containing the full name of the entity mentioned in the text so that the marked span starts and ends on a boundary between an alphanumeric string and a non-alphanumeric character (e.g. space or hyphen). The following provides examples and guidelines for exceptional cases.
- Modifiers and head words that are not part of the name are excluded from the annotated span. This includes the word “complex” which should not be annotated as part of the entity. For example in "human NFkappaB complex", only "NFkappaB" is annotated and in "phosphorylated RNA polymerase II" only "RNA polymerase II" is annotated.
```

- Affixes and markers are similarly excluded from the annotation span even when they are part of the same syntactic word with a Complex name as long as there is a separating nonalphanumeric character, for example in "phospho-RNA polymerase II" only "RNA polymerase II" is annotated.
- However, annotation boundaries must coincide with the boundary between an alphanumeric and a non-alphanumeric character. In cases where a Complex name is written with one of the following regular affixes without such a boundary, the affix is included in the annotated span: i) species identifier, e.g. h for human or m for mouse, ii) p for phosphorylated, iii) wt for wild-type, iv) si for small interfering RNA, v) sh for small/short hairpin RNA, vi) anti for antibody. For example in "the human complex hNFkappaB, and the murine complex mNFkappaB", "hNFkappa B" and "mNFkappaB" are annotated and in "pRNApolII (phosphor RNA polymerase II)", "pRNApolII" and "RNA polymerase II" are annotated.
- When a protein-containing complex name coincides with the name of a GGP or the GGPs comprising it or the name of a Protein Family, separated by ANY punctuation, annotation of the Protein or Protein Family named entities is preferred over annotation of the protein-containing complex entity. For example in "ERMES complex (otherwise known as Mdm10/Mdm12/Mmm1 complex)" only "ERMES" is annotated and "Mdm10/Mdm12/Mmm1" are not annotated as a single Complex entity, as they constitute three separate Protein NEs
- Two notable exceptions are "Arp2/3" and "SWI/SNF" where a single Protein-containing complex named entity is annotated instead.
- Annotations should be applied to all variants of a Complex name. For example "NF kappaB", "NF-kappaB", "NFkappaB" should all be marked as Protein-containing complex.
- PDB identifiers (for example "3BMP", "4BQ6") will NOT be annotated as Protein-containing complex even if when checked against PDB they correspond to one.
- List of GO terms which are protein-containing complex in GO, that will NOT be annotated as Complex as they consitute groups of complexes.
    * GO:1990104
    * GO:0140535
    * GO:1902494
    * GO:0070069
    * GO:0031074
    * GO:0005875
    * GO:0001114
    * GO:0150005
    * GO:0019907
    * GO:0044796
    * GO:0098636
    * GO:0098635
    * GO:0098637
    * GO:0043235
    * GO:0140368
    * GO:1903768
    * GO:0098666
    * GO:1990563
    * GO:0019036
    * GO:0098796
    * GO:0098803
    * GO:1990684
    * GO:1990686
    * GO:1990685
    * GO:0140513
    * GO:0035097
    * GO:0000109
    * GO:0008023
    * GO:0005849
    * GO:0000152

* GO:0032994
* GO:0060987
* GO:1990777
* GO:0030076
* GO:0098798
* GO:0090665
* GO:1990351
* GO:0005667
* GO:0017053
* GO:0090576
* GO:0090577
* GO:0090578
* GO:1903865
* GO:0000120
* GO:0090575
* GO:0032992
* GO:0046806
* GO:0032300
* GO:0032993
* GO:0031588
* GO:1990234
* GO:0061695
* GO:1902911
* GO:0009365
* GO:1902554
* GO:0000307
* GO:1902554
* GO:1902493
* GO:0031248
* GO:0031501
* GO:0106068
* GO:1902503
* GO:0042575
* GO:0030880
* GO:0005965
* GO:0034708
* GO:1990228
* GO:0016459
* GO:0045298
* GO:0031941
* GO:0005942
* GO:0000786
* GO:0038201
* GO:0034360
* GO:0071256
* GO:1990425
* GO:0034270
* GO:0106003

Supplementary Section 1.3: CoNECO GPT full - Results

Below are detailed results of the complexes that this custom version of ChatGPT has detected in the 25 documents provided as prompt. We used this list to count True Positives (TP), False Positives (FP) and False Negatives (FN) for each document and for the total set of documents.

PubMed ID	TP	FP	FN	detected complexes
10359895	0	6	0	YY1, Oct1
10660620	0	6	0	SHP-1
18342605	4	19	2	CPF, Glc7, Ref2, Swd2, Sen1, Nrd1-Nab3, Set1c
19608646	0	29	0	eNOS, CAV-1
21884581_16	0	5	0	B7.1-YPet, B7-H1-YPet, IR-YPet, B7-H1-Rluc8, B7.1-Rluc8
22723847_14	0	5	0	Ubp9, Ubp13, Duf1
23028367	5	0	0	Rpd3L, Rpd3S, Hda1
23427262_17	0	4	0	Dis1
26320581_12	0	7	0	MLL4, MLL1
7667268	0	27	3	TAFII80, TAFII31, TAFII250, TAFII20, TBP, TFIIE alpha, TFIIF alpha (RAP74)
7957109	10	10	4	NF-kappa B, I kappa B-alpha
8506307	0	9	0	gamma-carboxylase, PACE
9616112	0	2	0	CLOCK-BMAL1
11331609	1	3	0	NuRD, HDAC1, HDAC2
11359852	0	6	0	PLP
11675392	8	9	3	RabGGTase, REP-1
11832247	0	12	0	rab3b, pIgR
11917125	0	4	0	T1R2/T1R3, T1R1/T1R3
12022229	0	6	5	Nop7p
14592967	0	17	0	HSP90, RPM1, RAR1, SGT1
15220471	0	12	0	Sir2, Foxo1
16274718	0	3	0	ERalpha
17911105	3	16	0	gamma-secretase, presenilin, Pen-2, nicastrin, Aph-1
18253500	0	8	0	H1
9659924	1	0	6	IFN beta enhanceosome
Total	32	225	23	

Supplementary Section 1.4: CoNECO GPT small - Instructions

CoNECo GPT - small, available through this link: https://chat.openai.com/g/g-Ns0dcCn8c-coneco-gpt-small was created by providing the following set of instructions:

```
You are a helpful assistant. Your task is to identify protein-containing complexes
in the CoNECo corpus using the following annotation guidelines:
- Entities that fall within the scope of the Gene Ontology term protein-containing
complex are the target of named entity annotations for CoNECo: "A protein complex
in this context is meant as a stable set of interacting proteins which can be
co-purified by an acceptable method, and where the complex has been shown to exist
as an isolated, functional unit in vivo. Acceptable experimental methods include
stringent protein purification followed by detection of protein interaction. The
following methods should be considered non-acceptable: simple immunoprecipitation,
pull-down experiments from cell extracts without further purification,
colocalization and 2-hybrid screening. Interactions that should not be captured as
protein complexes include: 1) enzyme/substrate, receptor/ligand or any similar
transient interactions, unless these are a critical part of the complex assembly or
are required e.g. for the receptor to be functional; 2) proteins associated in a
pull-down/co-immunoprecipitation assay with no functional link or any evidence that
this is a defined biological entity rather than a loose-affinity complex; 3) any
complex where the only evidence is based on genetic interaction data; 4) partial
complexes, where some subunits (e.g. transmembrane ones) cannot be expressed as
recombinant proteins and are excluded from experiments (in this case, independent
evidence is necessary to find out the composition of the full complex, if known).
Interactions that may be captured as protein complexes include: 1) enzyme/substrate
or receptor/ligand if the complex can only assemble and become functional in the
presence of both classes of subunits; 2) complexes where one of the members has not
been shown to be physically linked to the other(s), but is a homologue of, and has
the same functionality as, a protein that has been experimentally demonstrated to
form a complex with the other member(s); 3) complexes whose existence is accepted
based on localization and pharmacological studies, but for which experimental
evidence is not yet available for the complex as a whole."
- If a term is found in Gene ontology but it is NOT a protein-containing complex,
then it will NOT be considered a Complex in this effort
- If a protein-containing complex can be identified as such by the annotators, but
there is no entry in Gene Ontology corresponding to the entity then it will receive
an annotation as a named entity (NE).
- The first resource that is trusted to resolve issues is Gene Ontology. If there
is still not enough information there, inconsistencies will be resolved using
Complex Portal, Reactome, CORUM, and as a last result the literature.
- For each mention of a protein-containing complex (Complex hereafter) name, the
annotation aims to mark the minimal span containing the full name of the entity
mentioned in the text so that the marked span starts and ends on a boundary between
an alphanumeric string and a non-alphanumeric character (e.g. space or hyphen).
- Modifiers and head words that are not part of the name are excluded from the
annotated span. This includes the word "complex" which should not be annotated as
part of the entity.
- Affixes and markers are similarly excluded from the annotation span even when
they are part of the same syntactic word with a Complex name as long as there is a
separating nonalphanumeric character.
- However, annotation boundaries must coincide with the boundary between an
alphanumeric and a non-alphanumeric character.
- When a protein-containing complex name coincides with the name of a Protein or
the Proteins comprising it or the name of a Protein Family, separated by ANY
punctuation, annotation of the Protein or Protein Family named entities is
preferred over annotation of the protein-containing complex entity.
- Two notable exceptions are "Arp2/3" and "SWI/SNF" where a single
Protein-containing complex named entity is annotated instead.
```

- Annotations should be applied to all variants of a Complex name.
- PDB identifiers will NOT be annotated as Protein-containing complex.
- GO terms which are protein-containing complex in GO, but consitute groups of complexes, will not be annotated as Complex. This includes GO terms the definition of which starts with the phrase: "A protein complex which is capable of...".

Supplementary Section 1.5: CoNECO GPT small - Results

Below are detailed results of the complexes that this custom version of ChatGPT has detected in the 25 documents provided as prompt. We once again used this list to count TP, FP and FN for each document and for the total set of documents.

PubMed ID	TP	FP	FN	detected complexes
10359895	0	6	0	YY1, Oct1
10660620	0	0	0	-
18342605	4	2	0	CPF, Nrd1-Nab3, Set1c
19608646	0	29	0	eNOS, CAV-1
21884581_16	0	5	0	B7.1-YPet, B7-H1-YPet, IR-YPet, B7-H1-Rluc8, B7.1-Rluc8
22723847_14	0	0	0	-
23028367	5	0	0	Rpd3L, Rpd3S, Hda1
23427262_17	0	4	0	Dis1
26320581_12	0	7	0	MLL4, MLL1
7667268	3	27	0	TFIID,TAFII80,TAFII250,TAFII31,TAFII20,TBP,TFIIE alpha,TFIIF alpha
7957109	10	10	4	NF-kappa B,I kappa B-alpha
8506307	0	9	0	gamma-carboxylase, PACE
9616112	0	2	0	CLOCK-BMAL1
11331609	1	2	0	NuRD, SRA
11359852	0	0	0	-
11675392	8	7	3	RabGGTase, REP-1, RabGGTase.GGpp.REP-1
11832247	0	0	0	-
11917125	0	4	0	T1R2/T1R3, T1R1/T1R3
12022229	0	6	5	Nop7p
14592967	0	17	0	HSP90, RPM1, RAR1, SGT1
15220471	0	12	0	Foxo1,Sir2
16274718	0	3	0	ERalpha
17911105	3	0	0	gamma-secretase
18253500	0	0	0	-
9659924	3	5	0	CBP/p300, IFN beta enhanceosome, NF-kappa B, IRF1, ATF2/c-Jun, HMG I(Y)
Total	37	157	12	

Supplementary Section 1.6: CoNECO GPT minimal - Instructions

“CoNECo GPT - minimal” is available through the Explore GPTs section of ChatGPT4.0 https://chat.openai.com/g/g-C6Nx12aEL-coneco-gpt-minimal and was generated using the instructions below:

```
Coneco specializes in the identification of protein complexes with unwavering adherence to a set of precise guidelines. It defines protein complexes based on the Gene Ontology term 'protein-containing complex' (GO:0032991) within the cellular component. A protein complex is identified as a stable set of interacting proteins that are co-purifiable and function together in vivo. Methods like stringent protein purification followed by interaction detection are acceptable for validation, but not mere immunoprecipitation or colocalization. Entities not categorized as 'protein-containing complex' in Gene Ontology or not children of GO:0032991, including entities such as ribosomes, are not considered protein complexes by Coneco. In its identification process, Coneco first verifies if an entity is a protein using UniProt, then checks if it's a protein family in InterPro. Entities that are neither proteins nor protein families are then evaluated against Coneco's rules, referencing Gene Ontology, Complex Portal, Reactome, CORUM, and relevant literature. Coneco annotates minimal spans of protein complex names, avoiding non-essential modifiers. Entities coinciding with genes, gene products, or protein families, without evidence of being a functional entity, are not annotated as complexes. Additionally, certain GO-classified protein-containing complexes are excluded from annotation. Coneco has two specific rules to enhance its accuracy: 1) If entities are separated by a dash (-), they are examined and reported separately, with each being individually assessed against the established guidelines. 2) If terms examined are proteins or protein families, they are not reported as protein complexes. Coneco relies on resources like PubMed, GO, InterPro, and UniProt for verification, strictly following these guidelines and definitions.
```

Supplementary Section 1.7: CoNECO GPT minimal - Results

The results for the same set of 25 prompts are the following:

PubMed ID	TP	FP	FN	detected words
10359895	0	0	0	-
10660620	0	0	0	-
18342605	2	2	4	cleavage and polyadenylation factor, Set1c histone H3K4 methyltransferase complex, Nrd1-Nab3 complex
19608646	0	0	0	-
21884581_16	0	0	0	-
22723847_14	0	0	0	-
23028367	5	0	0	Rpd3L, Rpd3S, Hda1 complexes
23427262_17	0	0	0	-
26320581_12	0	0	0	-
7667268	3	0	0	TFIID
7957109	10	0	4	NF-kappa B/I kappa B-alpha complex
8506307	0	0	0	-
9616112	0	1	0	CLOCK-BMAL1 heterodimers
11331609	1	0	0	NuRD complex
11359852	0	0	0	-
11675392	2	0	9	RabGGTase.GGpp.REP-1 complex
11832247	0	0	0	-
11917125	0	4	0	T1R2/T1R3, T1R1/T1R3
12022229	2	0	3	pre-60S particles
14592967	0	0	0	-
15220471	0	0	0	-
16274718	0	0	0	-
17911105	3	0	0	gamma-secretase complex
18253500	0	0	0	-
9659924	1	0	6	IFN beta enhanceosome
Total	29	7	26	
